# Spoofing Detection Using GNSS/INS/Odometer Coupling for Vehicular Navigation

**DOI:** 10.3390/s18051305

**Published:** 2018-04-24

**Authors:** Ali Broumandan, Gérard Lachapelle

**Affiliations:** Position, Location and Navigation (PLAN) Group, Schulich School of Engineering, University of Calgary, Calgary, AB T2N 1N4, Canada; lachapel@ucalgary.ca

**Keywords:** global navigation satellite systems (GNSS), spoofing, detection, IMU, vehicular navigation

## Abstract

Location information is one of the most vital information required to achieve intelligent and context-aware capability for various applications such as driverless cars. However, related security and privacy threats are a major holdback. With increasing focus on using Global Navigation Satellite Systems (GNSS) for autonomous navigation and related applications, it is important to provide robust navigation solutions, yet signal spoofing for illegal or covert transportation and misleading receiver timing is increasing and now frequent. Hence, detection and mitigation of spoofing attacks has become an important topic. Several contributions on spoofing detection have been made, focusing on different layers of a GNSS receiver. This paper focuses on spoofing detection utilizing self-contained sensors, namely inertial measurement units (IMUs) and vehicle odometer outputs. A spoofing detection approach based on a consistency check between GNSS and IMU/odometer mechanization is proposed. To detect a spoofing attack, the method analyses GNSS and IMU/odometer measurements independently during a pre-selected observation window and cross checks the solutions provided by GNSS and inertial navigation solution (INS)/odometer mechanization. The performance of the proposed method is verified in real vehicular environments. Mean spoofing detection time and detection performance in terms of receiver operation characteristics (ROC) in sub-urban and dense urban environments are evaluated.

## 1. Introduction

Spoofing signals are designed to mislead GNSS receivers by generating fabricated synchronized navigation signals leading to fake navigation solutions [1,2,3]. Hence, detection and mitigation of spoofing attacks is critical for emerging applications such as autonomous vehicle navigation, environmental monitoring and forensic applications [4,5]. Several related contributions have been made with focus on different layers of the receiver including antenna, IF samples, acquisition, tracking and navigation [6,7,8,9,10,11,12]. The spoofing detection techniques implemented in the pre-despreading and signal processing layers of a receiver are effective and can detect spoofing attacks faster than the methods implemented in the navigation layer [13]. However, these techniques require several modifications to current receiver designs. Several spoofing detection methods implemented in the measurement and navigation layers have been proposed. For example, Reference [11] implemented a position solution authenticity verification technique based on clock bias variation analysis of a moving receiver. A spoofing detection metric using carrier phase measurements with multiple receivers was implemented in [12].

In addition to the above standalone approaches, spoofing attacks can be detected by checking the consistency of the navigation solutions under test with other reference sources [13,14]. Consistency checks can be performed in different ways including intra-system, inter-system, multi-frequency and multi-sensor approaches. In the intra-system consistency check, the presence of spoofing signals can be detected by monitoring the consistency of the code and carrier Doppler or by monitoring the carrier-to-noise ratio [1,13]. The emergence of different civilian GNSS constellations has led to the availability of multi-constellation receivers. Such a receiver can be designed to perform various inter-system cross-checks among different signal ensembles in order to verify the authenticity of received signal sets [14]. Modernized GNSS systems transmit civilian signals in different frequency bands. From a spoofer’s viewpoint, it is considerably more difficult/costly to simultaneously spoof many frequency bands. Therefore, a multi-frequency receiver can perform some cross checks to verify the authenticity of received signal sets. Augmenting data with auxiliary devices such as IMUs can help the target receiver to discriminate against the spoofing threat [15,16,17,18,19,20,21,22,23]. In addition, a receiver can compare the solution extracted from received signals to position and navigation solutions obtained by other means, e.g., mobile networks or Wi-Fi access points [15]. Therefore, if the confidence region of different solutions does not have an intersection, there is a high likelihood of a spoofing attack. Another spoofing scenario rarely discussed in the literature is the case when the spoofer has access to the GNSS receiver antenna and may deny authentic signal reception by covering the antenna and feeding spoofed signals. In such a case, most proposed spoofing detection methods in various signal processing layers of a receiver are not functional.

GNSS and inertial navigation systems (INS) have complementary error characteristics: GNSS has good long-term accuracy whereas INS has good short term accuracy. INS is self-contained, operates continuously and provides navigation solutions with low short-term noise. However, it suffers from accuracy degradation over time due to the integration of biases and drifts of the inertial measurement units (IMUs). Along with navigation solutions, attitude information can also be estimated from the INS, which is important for many applications. The complementary features of GNSS and INS make them a good choice for integration especially when GNSS observability is poor. Advantages and performance of loosely, tightly and ultra-tightly coupled GNSS/INS integration methods have been studied and reported by a number of researchers [24,25,26]. In a typical GNSS-INS system, an IMU with three orthogonally mounted accelerometers and three gyroscopes is used. For land based vehicular applications, to reduce the cost associated with a full INS, a reduced number of sensors can be used, also known as a reduced inertial Sensor system (RISS) [27,28,29]. Even though integration of GNSS and INS provides robust navigation, performance will be degraded under spoofing attacks. Under such attacks, GNSS measurements will be erroneous which in turn makes the integrated solution unreliable.

In an integrated GNSS-INS system, since only GNSS measurements are potentially erroneous due to spoofing, INS measurements can be used in an integrity monitoring role to detect an attack. Authors in [19] proposed an integrated GPS/INS navigation system to detect a spoofing attack based on the receiver autonomous integrity monitoring (RAIM) concept. The integrity risk has been evaluated in the presence of high-end and low-end INS systems and it is shown that the proposed approach is able to successfully detect spoofing attacks that do not have previous knowledge of the receiver’s trajectory. A shipboard IMU measurements in [18] was used to detect the presence of spoofing signals; their approach compares the relative platform motion estimates provided by a shipboard receiver to the ones provided by the onboard IMU. It was shown that high frequency pitch/roll motion of the ship caused by mild sea conditions can lead to successful spoofing detection. In [17] a tightly coupled GNSS/INS approach to detect spoofing signals was proposed. The method detects spoofing attacks by monitoring the residuals and sets the spoofing detection based on minimum detectable blunder test statistics. It assumes that a subset of visible PRNs is spoofed at a given time which limits its practicality.

Herein, a spoofing detection approach based on a consistency check of GNSS and INS/odometer (odo) mechanization is proposed. To detect a spoofing attack, the proposed method analyzes GNSS and IMU/odo measurements during an observation window and compares the two solutions (trajectories). The INS-vehicle odometer integrated solution is self-contained and therefore not vulnerable to external signal jamming and spoofing. However, like all dead reckoning devices, it is susceptible to sensor induced errors, especially drift. The spoofing detection observation window is defined based on the INS characteristics and the specific application. GNSS signal authenticity is verified if its navigation solution is consistent with the corresponding solution provided by INS/odo. If GNSS signal authenticity is verified, the GNSS/INS/odometer loose coupling solution is performed to estimate and remove IMU errors.

## 2. GNSS Spoofing Detection Using IMU and Odometer

INS and their solutions are self-contained and provide high rate measurements. They have good short-term accuracy. However, long term errors grow without bound as the inertial sensor errors accumulate due to intrinsic integration in the navigation algorithm. Navigation solutions based on GNSS need a direct line of sight to at least four satellites, which is not always possible due to satellite signal blockages by tall buildings, trees and tunnel entrances and exits. Taking advantage of the complementary characteristics of these systems, their integration overcomes their individual drawbacks and provides a more accurate and robust navigation solution than neither could achieve. The integrated navigation solution is a continuous high data rate system that provides a full navigation solution (position, velocity and attitude) with improved accuracy in both the short and long term. GNSS prevents the inertial solution from drifting and INS provides continuity in the navigational solution. In the loosely coupled integration case, GNSS navigation solutions and INS mechanization operate independently and provide separate navigation solutions. To improve the solution, the position and/or velocity from GNSS is fed to an optimal estimator, usually a Kalman Filter (KF). The INS solution is also supplied to the filter, which takes the difference between the two and, based upon the error models, estimates the INS errors. In general, two types of feedback approaches are implemented, namely open-loop and closed-loop. In the open-loop configuration, the position, velocity and attitude corrections are performed in the integrated navigation solution (external to the INS) where the estimated errors are subtracted from the INS solution at each iteration. In such a case, the corrected KF states are not fed back to the INS to correct for its drift. In the closed-loop configuration, the error estimates from KF are fed back in order to correct the INS errors. The output of the INS forms the integrated solution. KF position, velocity and attitude estimates are reset to zero after the error estimates are fed back. In the conventional implementation of GNSS/INS, the integration filter runs in prediction mode with the predicted values of the INS during the GNSS outages. In open sky conditions when the receiver antenna has access to Line-Of-Sight (LOS) signals, either the integrated or the unaided GNSS solution can be used. More specifically, under nominal operation conditions the signal and measurement quality of GNSS are high and the KF puts more weight on the GNSS measurements than on prediction. As mentioned previously, the integration of GNSS/INS is beneficial in GNSS outage scenarios. However, in the case of a spoofing attack the reasonable assumption is that the receiver antenna receives spoofed GNSS signals with a high signal strength, resulting in a fake navigation solution. In such a case, GNSS/INS integration under a closed loop scenario with an update rate of a few Hz will not be effective in detecting the spoofing attack. This is due to the fact that in the closed-loop integration, the integrated KF solution’s estimated accelerometer and gyroscope errors are fed back to correct the IMU measurements. These errors are applied on every iteration of mechanization, with feedback from KF periodically updating the accelerometer and gyroscope errors. Since the relative dynamics between spoofed GNSS solutions and that of INS are probably not significant for a vehicle during a typically short update interval (a few Hz), the spoofing attack may not be detected.

The advantage of the open loop configuration is that in addition to the integrated navigation solution, the raw INS solution can support integrity monitoring and spoofing detection since the inertial based navigation solutions are not affected by the attack. However, due to INS drift, the errors in the INS grow with time to the point that the authenticity verification using this approach is no longer reliable. Considering this, a possible approach to detect the spoofing attack and enhance the performance of the authenticity verification procedure is to use a closed loop configuration with a shorter error feedback update rate. In such a case, the receiver will operate under normal conditions and an additional loop will monitor the integrity of the solution. The integrity monitoring loop takes raw IMU measurements and provides navigation solutions without the error correction from GNSS measurements. In this case, the integrity monitoring loop error correction update rate is much smaller than that of the KF integration update rate. The update rate of the integrity monitoring is based on the IMU grade and specific application requirements. To avoid false spoofing detection due to IMU drift, it is important to characterize the performance of the authenticity verification loop during the observation interval and set a proper detection threshold.

**Figure 1:**
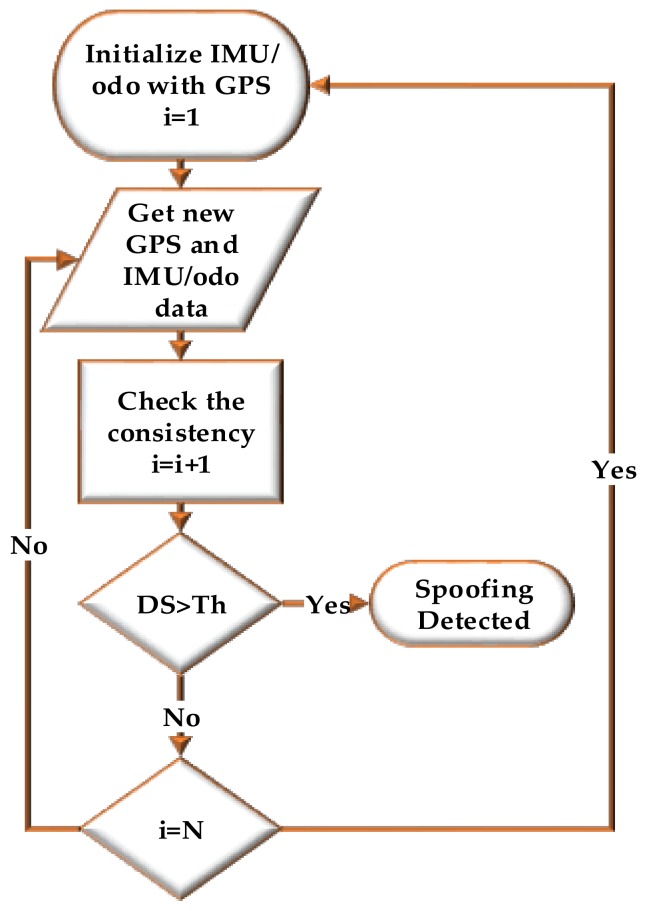
Operational flowchart of the proposed method.

A spoofing detection approach based on a consistency check of GNSS and INS/odometer (odo) mechanization is now proposed. To detect a spoofing attack, the method analyzes GNSS and IMU/odo measurements during an observation window and compares the solution provided by GNSS and INS mechanizations. The two trajectories are compared and the Detection Statistic (*DS*) is calculated as:

(1) $$DS=\left\|p_{k}^{GPS}-p_{k}^{IMU/odo}\right\|,\begin{array}{cc} & p=\left[\begin{array}{l} p_{E}\\ p_{N}\\ p_{U} \end{array}\right] \end{array}$$

where $p_{k}^{GPS}$ and $p_{k}^{IMU/odo}$ are GPS and IMU/odo position vectors at time *k* in the East-North-Up (ENU) frame and ‖ ‖ is the norm operator. GNSS signal authenticity is verified if DS is below a predefined threshold. The detection threshold is based on the INS/odo characteristics and the specific application and should be determined based on a desired probability of false alarm under authentic signal operation condition. If the signal authenticity is verified, a GNSS/INS/odometer loose coupling solution is performed to remove IMU errors. Figure 1 shows the operation flowchart of the proposed spoofing detection method. The IMU/odo mechanization process is initialized with the GPS measurements. Then the consistency of a new set of GPS and IMU/odo measurements is analysed. If the detection statistics (*DS*) is above the threshold, a spoofing attack is detected. Otherwise, the detection process checks the length of IMU/odo data (*i*) processed without correction by GPS measurements. If *i* is less than the length of the observation window (*N*) then consistency of a new GPS and IMU/odo data is processed. When *i* = *N* and no spoofing is edetected, the monitoring loop updates the IMU and odo errors with GPS measurements. 

**Figure 2:**
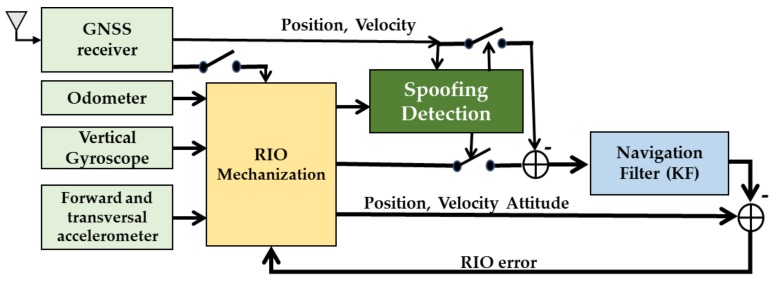
Loosely coupled RIO mechanization for spoofing detection.

Reduced IMU and odometer (RIO) mechanization, which is suitable for any wheel-based platform, is considered for GNSS navigation solution authenticity verification. RIO mechanization eliminates several error sources that exist when using a full IMU, especially low-cost Micro-Electro-Mechanical Systems (MEMS) grade sensors, and consequently reduces navigation solution divergence during GNSS outages and enhances the performance of the authenticity verification procedure. The significance and the importance of the RIO solution over the full IMU is discussed in [27]. Figure 2 shows the authenticity verification loop considering RIO mechanization with loosely coupled RIO/GNSS integration. The forward velocity information along with raw accelerometer and gyroscope measurements are fed to the RIO mechanization to provide relative position, velocity and heading information. The authenticity verification unit compares the navigation solution of the GNSS with that of RIO during the observation interval. If the solution authenticity is verified. RIO solutions will be corrected by GNSS solutions and accelerometer and gyro errors will be corrected by the navigation KF.

## 3. RIO Mechanization

RIO mechanization and the loose coupling model used are described in this section. The local-level frame is the East-North-Up (ENU) frame with axes along east, north and vertical (up) directions. The sensors measurements provided by the gyroscope, the two accelerometers and the odometer comprise the control inputs represented by the vector:

(2) $$u_{i}=\left[v_{i}^{o},a_{i}^{o},f_{i}^{x},f_{i}^{y},\omega_{i}^{z}\right]{\;}^{T}$$

where $v_{i}^{o}$ is the speed from the odometer output, $a_{i}^{o}$ is the acceleration from the vehicle odometer output, $f_{i}^{x}$ and $f_{i}^{y}$ are the *x* and *y* accelerometer outputs and $\omega_{i}^{z}$ the vertical gyroscope output. The vertical gyroscope is mounted in alignment with the vertical axis of the vehicle and two accelerometers are mounted in the transversal and forward directions. The rate gyroscope is used to measure heading change of the vehicle and two accelerometers to measure changes in roll and pitch of the vehicle. The vehicle attitude information along with odometer derived forward speed are used to compute the user velocities in the ENU frame. Subsequently, the user position is obtained by integrating the velocity solution.

The state vector for the mechanization is:

(3) $$x_{k}=\left[\varphi_{k},\lambda_{k},h_{k},v_{k}^{E},v_{k}^{N},v_{k}^{U},p_{k},r_{k},A_{k}\right]{\;}^{T}$$

where $\;\left\{\varphi_{k},\lambda_{k},h\right\}$ is the position vector in the geodetic coordinate frame, $\left\{v_{k}^{E},v_{k}^{N},v_{k}^{U}\right\}$ is the velocity vector in the East-North-Up (ENU) coordinate frame, and $\left\{p_{k},r_{k},A_{k}\right\}$ is the pitch, roll and azimuth angles of the vehicle. The pitch angle is computed from the forward accelerometer. When the vehicle is in motion, the accelerometer measures the forward acceleration as well as the component due to gravity. In order to compute the pitch angle, this forward acceleration needs to be removed from the forward accelerometer measurement. Similarly, for roll angle computation, the transversal accelerometer measurement needs to be compensated with the normal component of the acceleration. The azimuth angle is computed from the vertical gyroscope and its measurement is compensated with earth rotation as well as the rotation of the local level frame with earth’s curvature. Thus, the mechanization equations to compute the vehicle attitude information is given by:

(4) $$\begin{array}{l} r_{i}=-\sin^{-1}\left(\frac{f_{i}^{x}+v_{i}^{o}\omega_{i}^{z}}{g\cos p_{i}}\right)\\ p_{i}=\sin^{-1}\left(\frac{f_{i}^{y}-a_{i}^{o}}{g}\right)\\ A_{i}=A_{i-1}-\omega_{i}^{z}\Delta t+\omega^{e}\sin\varphi_{i-1}\Delta t+\frac{v_{i-1}^{E}\tan\varphi_{i-1}}{R_{N}+h_{i-1}}\Delta t \end{array}$$

where r,p,A are the roll, pitch and heading of the vehicle, and g is gravity. The user velocity in ENU frame can be obtained as:

(5) $$\begin{array}{l} v_{i}^{E}=v_{i}^{o}\sin A_{i}\cos p_{i}\\ v_{i}^{N}=v_{i}^{o}\cos A_{i}\cos p_{i}\\ v_{i}^{U}=v_{i}^{o}\sin p_{i} \end{array}$$

User coordinates can be obtained as:

(6) $$\begin{array}{l} \varphi_{i}=\varphi_{i-1}+\frac{v_{i}^{N}}{R_{M}+h_{i}}\Delta t\\ \lambda_{i}=\lambda_{i-1}+\frac{v_{i}^{E}}{\left(R_{N}+h_{i}\right)\cos\varphi_{i}}\Delta t\\ h_{i}=h_{i-1}+v_{i}^{U}\Delta t \end{array}$$

where φ, λ and h are latitude, longitude and height. In the loose coupling approach, GNSS and RIO navigation solutions are combined in a navigation KF. Both system and measurement model are nonlinear. Since linearization is performed, only the perturbations in the states are computed in the filter. The linearized discrete system model is given by:

(7) $$\delta x_{k}=\Phi_{k-1}\delta x_{k-1}+G_{k-1}W_{k-1}$$

where $\delta x_{k}$ is the 9 × 1 error state vector at time epoch *k* given by $\delta x_{k}=\left\{\delta \phi_{k},\delta \lambda_{k},\delta h_{k},\delta v_{k}^{E},\delta v_{k}^{N},\delta v_{k}^{U},\delta A_{k}^{},\delta S_{k}^{od},\delta \omega_{k}^{z}\right\}$.

$\delta \phi_{k},\delta \lambda_{k},\delta h_{k}$ are the position vector components in the geodetic coordinate frame, $\delta v_{k}^{E},\delta v_{k}^{N},\delta v_{k}^{U}$ are the velocity vectors in East-North-Up (ENU) coordinate frame, $\delta A_{k}$ is the azimuth angle, $\delta S_{k}^{od}$ is the scale factor of odometer, $\delta \omega_{k}^{z}$ is the vertical gyroscope drift, $\Phi_{k-1}$ is the state transition matrix from time epoch *k* − 1 to *k*, $G_{k-1}$ is the shaping matrix or noise coupling matrix and $W_{k-1}$ the zero mean unity variance white noise.

The linearized discrete system model is given by:

(8) $$\delta x_{k}=\left[\begin{array}{l} \delta p_{k}\\ \delta v_{k}\\ \delta e_{k} \end{array}\right]=\left[\begin{array}{ccc} \begin{array}{l} I_{3\times 3}\\ 0_{3\times 3}\\ 0_{3\times 3} \end{array} & \begin{array}{l} F_{1}\\ I_{3\times 3}\\ 0_{3\times 3} \end{array} & \begin{array}{l} 0_{3\times 3}\\ F_{2}\\ F_{3} \end{array} \end{array}\right]\left[\begin{array}{l} \delta p_{k-1}\\ \delta v_{k-1}\\ \delta e_{k-1} \end{array}\right]+\left[\begin{array}{l} 0_{3\times 1}\\ 0_{3\times 1}\\ \delta \sigma_{k} \end{array}\right]$$

where:

$$\delta p_{k}=\left[\begin{array}{l} \delta \phi_{k}\\ \delta \lambda_{k}\\ \delta h_{k} \end{array}\right],\;\delta r_{k}=\left[\begin{array}{l} \delta v_{k}^{E}\\ \delta v_{k}^{N}\\ \delta v_{k}^{U} \end{array}\right],\;\delta e_{k}=\left[\begin{array}{l} \delta A_{k}^{}\\ \delta S_{k}^{od}\\ \delta \omega_{k}^{z} \end{array}\right],\;\delta \sigma_{k}=\left[\begin{array}{l} 0\\ \sqrt{2\gamma_{od}\sigma_{od}^{2}\Delta t}\\ \sqrt{2\gamma_{\omega z}\sigma_{\omega z}^{2}\Delta t} \end{array}\right],$$

$$F_{1}=\left[\begin{array}{ccc} 0 & \frac{\Delta t}{R_{m}+h_{k-1}} & 0\\ \frac{\Delta t}{\left(R_{n}+h_{k-1}\right)\cos\left(\phi_{k-1}\right)} & 0 & 0\\ 0 & 0 & \Delta t \end{array}\right]$$

$$F_{2}=\left[\begin{array}{ccc} v_{od}\cos\left(A_{k-1}\right)\cos\left(p_{k-1}\right)\Delta t & v_{od}\sin\left(A_{k-1}\right)\cos\left(p_{k-1}\right)\Delta t & 0\\ v_{od}\sin\left(A_{k-1}\right)\cos\left(p_{k-1}\right)\Delta t & v_{od}\cos\left(A_{k-1}\right)\cos\left(p_{k-1}\right)\Delta t & 0\\ 0 & v_{od}\sin\left(p_{k-1}\right)\Delta t & 0 \end{array}\right]$$

$$F_{3}=\left[\begin{array}{ccc} 1 & 0 & -\Delta t\\ 1 & 1-\gamma_{od}\Delta t & 0\\ 0 & 0 & 1-\gamma_{\omega z}\Delta t \end{array}\right]$$

where $\gamma_{od}$ and $\gamma_{\omega z}$ are the inverse of autocorrelation time for odometer and gyroscope stochastic errors, $\sigma_{od}^{2}$ and $\sigma_{\omega z}^{2}$ are the variance of odometer and gyroscope noise [26]. The linearized discrete measurement model is given by:

(9) $$\delta z_{k}=H\delta x_{k}+\varepsilon_{k}$$

where $\delta z_{k}$ is the measurement vector given by:

(10) $$\delta z_{k}=\left[\begin{array}{l} \phi_{k}^{GPS}-\phi_{k}^{RIO}\\ \lambda_{k}^{GPS}-\lambda_{k}^{RIO}\\ h_{k}^{GPS}-h_{k}^{RIO}\\ v_{k}^{E,GPS}-v_{k}^{E,RIO}\\ v_{k}^{N,GPS}-v_{k}^{N,RIO}\\ v_{k}^{U,GPS}-v_{k}^{U,RIO} \end{array}\right]$$

H is the design matrix and $\varepsilon_{k}$ represents measurement noise.

The design matrix is given by:

(11) $$H=\left[\begin{array}{ccccccccc} \begin{array}{l} 1\\ 0\\ 0\\ 0\\ 0\\ 0 \end{array} & \begin{array}{l} 0\\ 1\\ 0\\ 0\\ 0\\ 0 \end{array} & \begin{array}{l} 0\\ 0\\ 1\\ 0\\ 0\\ 0 \end{array} & \begin{array}{l} 0\\ 0\\ 0\\ 1\\ 0\\ 0 \end{array} & \begin{array}{l} 0\\ 0\\ 0\\ 0\\ 1\\ 0 \end{array} & \begin{array}{l} 0\\ 0\\ 0\\ 0\\ 0\\ 1 \end{array} & \begin{array}{l} 0\\ 0\\ 0\\ 0\\ 0\\ 0 \end{array} & \begin{array}{l} 0\\ 0\\ 0\\ 0\\ 0\\ 0 \end{array} & \begin{array}{l} 0\\ 0\\ 0\\ 0\\ 0\\ 0 \end{array} \end{array}\right]$$

## 4. Data Collection Scenarios

**Figure 3:**
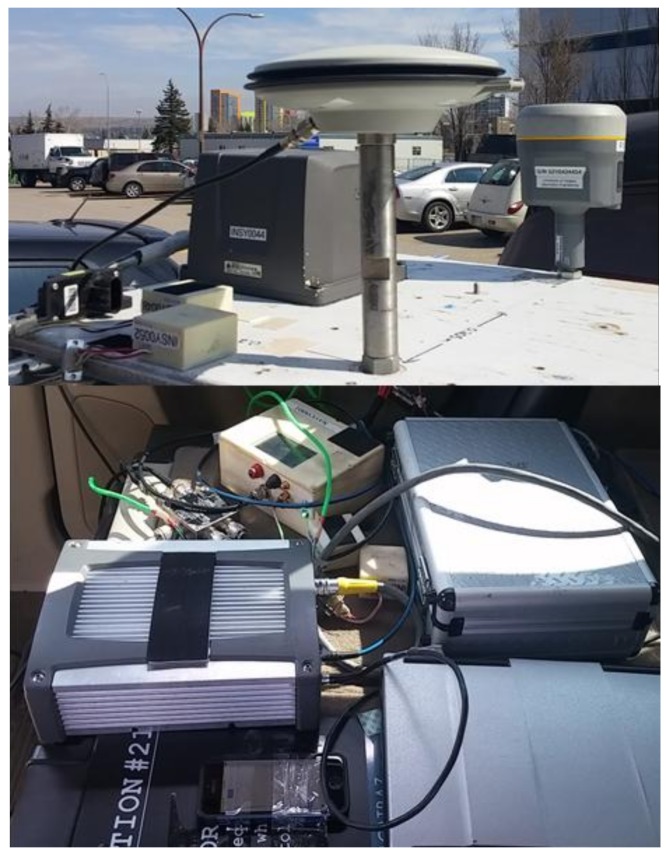
Data collection setup.

Actual GPS and IMU data was collected using a vehicle in urban and suburban areas of Calgary. The experimental setup used is shown in Figure 3. Data was collected using tactical and MEMS grade IMUs whose specifications are given in Table 1. A navigation grade GNSS antenna was mounted on the vehicle roof and GNSS signals were passed to a two-way splitter. One branch was connected to a SPAN/LCI GNSS/INS system (NovAtel, Calgary, Alberta, AB, Canada) using the NovAtel’s Inertial Explorer™ software in dual-frequency RTK mode with forward and backward smoothing to provide a sub-metre reference trajectory. The other branch was connected to a u-blox (EVK-6) receiver to provide GPS measurements. The IMUs used consist of 3-axis accelerometers and rate gyroscopes orthogonally mounted to each other.

The *z* gyroscope was aligned with the vertical axis of the vehicle body frame and used for computing the azimuth angle. The *x* and *y* accelerometers were aligned with the forward and transversal axes of the vehicle body frame and used to calculate pitch and roll. The inertial sensors measurements are in the body frame. A rotation matrix from the body frame to the local level frame was estimated at the beginning. An on-board diagnostics (OBD) device was connected to the car OBD port and used to collect vehicle speed via a LG G3 cell phone. A cell phone application named Torque was used to collect vehicle speed. During the data collection process, the cell phone’s various sensor outputs including GNSS navigation solutions and raw IMU values were also logged. The cell phone containing the MEMS IMU was installed on the back seat below the tactical IMU which was mounted on the vehicle roof.

## 5. Spoofing Detection Performance

The proposed spoofing detection performance is evaluated by comparing the reduced-inertial and odometer (RIO) trajectory to that of the GPS-based trajectory. An open loop structure is used herein and results in three solutions computed in parallel, namely GPS, RIO and GPS/RIO as discussed in the sequel. In the GPS/RIO solution, the RIO errors at each update interval are corrected using actual GPS measurements. Hence, in the presence of a spoofing attack, the GPS/RIO integration can be easily spoofed. Comparing the GPS solutions with the RIO solutions over an extended interval is not practical due to RIO initial errors and accumulated sensor drifts. Therefore, for GPS authenticity verification purpose, the RIO correction update rate with GPS is adjusted as a function of the application. Between RIO updates, the RIO position errors are therefore bounded and are not contaminated by spoofed GPS measurements. To detect GPS signal spoofing, the trajectory provided by the receiver is compared to that of the RIO trajectory over the intervals between updates. Spoofing is detected if the difference is higher than a predefined threshold. Two data sets (Set 1 and 2) collected in suburban and dense urban environments to test the above approach are analysed below to demonstrate the performance of the approach.

### 5.1. Results of Data Set 1 (Suburban Environment)

**Figure 4:**
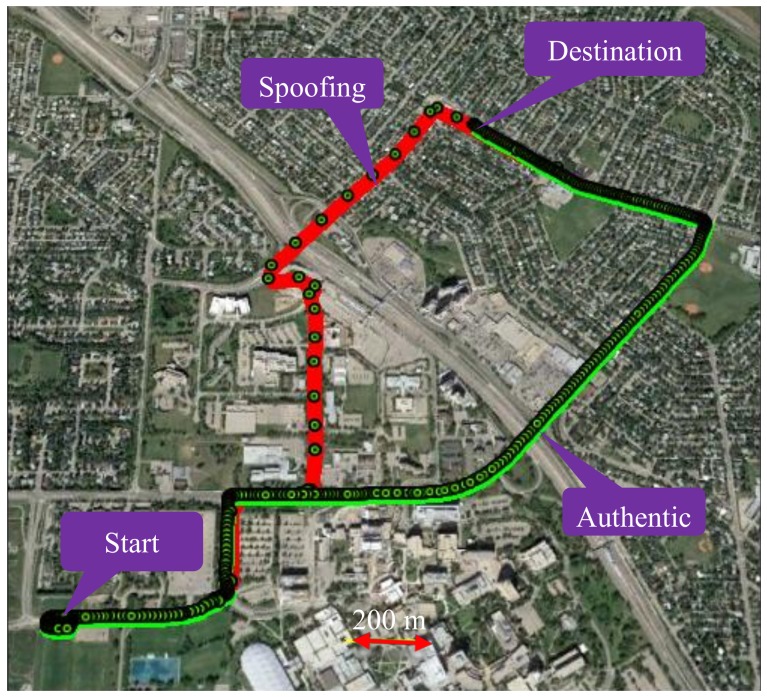
Data collection scenario in suburban environment. Green and red lines are the authentic and spoofed trajectories.

Figure 4 shows trajectories in a suburban residential environment. To emulate a spoofing attack, the red trajectory, driven first with only a GPS receiver, is assumed to be the spoofed one. The GPS measurements of that trajectory are then used when driving the authentic green trajectory with the tactical and MEMS IMUs and use of vehicle odometer. Both trajectories started at the same location and reached the same destination from the two paths shown. The two trajectories overlapped in some parts of the initial path and then separated and joined again at destination. The two IMUs, odometer and GPS receiver logged data simultaneously. A spoofing attack is detected when the GPS red spoofed trajectory does not match that of the RIO trajectory on the green authentic trajectory during the detection observation intervals and threshold selected as described below.

**Figure 5:**
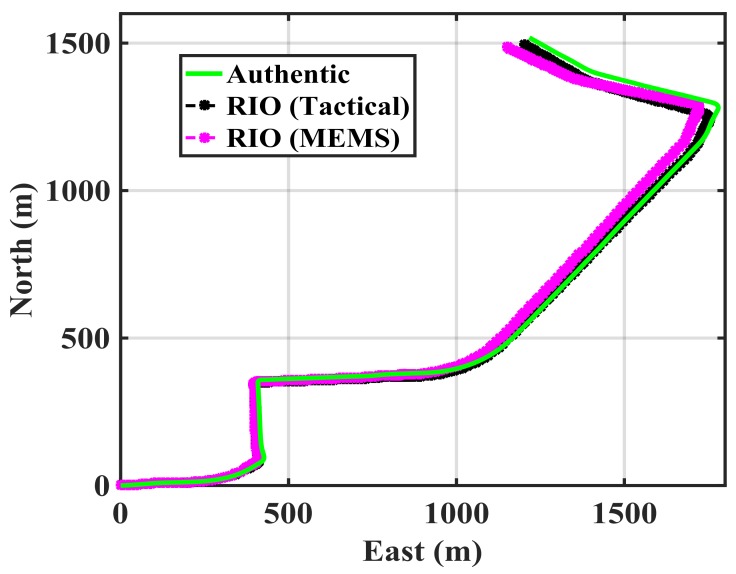
Standalone RIO mechanization for the two different IMUs used (350 s of data).

**Figure 6:**
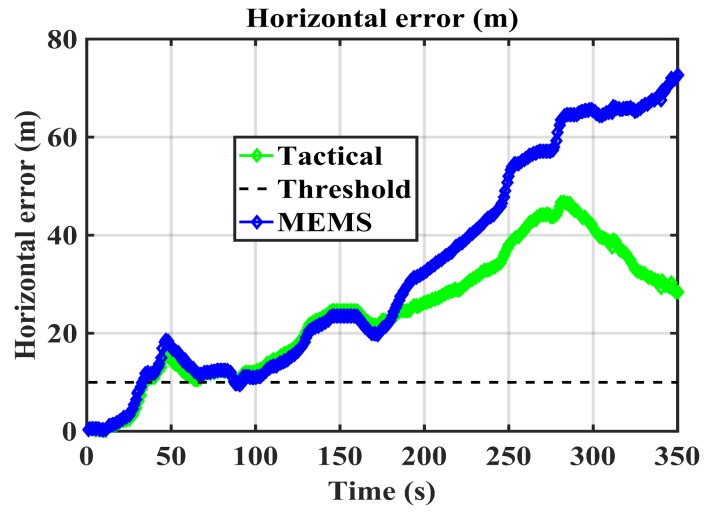
RIO mechnization errors for the two different IMUs shown in Figure 4.

The first step to assess the performance of the method is to evaluate its characteristics under a null-hypothesis (absence of spoofing attack). This means evaluating the performance of the RIO for each IMU configuration. Figure 5 shows the authentic trajectory in green as obtained by spoof-free GPS and each of the RIO trajectories over the 350 s duration of the test. The GPS-free RIO trajectories were initialized with correct position and heading information. The RIO solutions generally follow the authentic trajectory pattern, however, the errors in the trajectory estimation differ for the two IMUs as expected. Figure 6 shows their growing horizontal errors as a function of time and obtained through a comparison with the spoof-free GPS trajectory. As expected, the RIO solution using the tactical grade IMU results in better performance and maintains a horizontal accuracy better than 50 m. The performance of the cell phone MEMS IMU-based RIO is comparable to that of the tactical one only for the initial 150 s of data. The spoofing detection threshold of 10 m selected is arbitrary and can be adjusted as required by the application.

**Figure 7:**
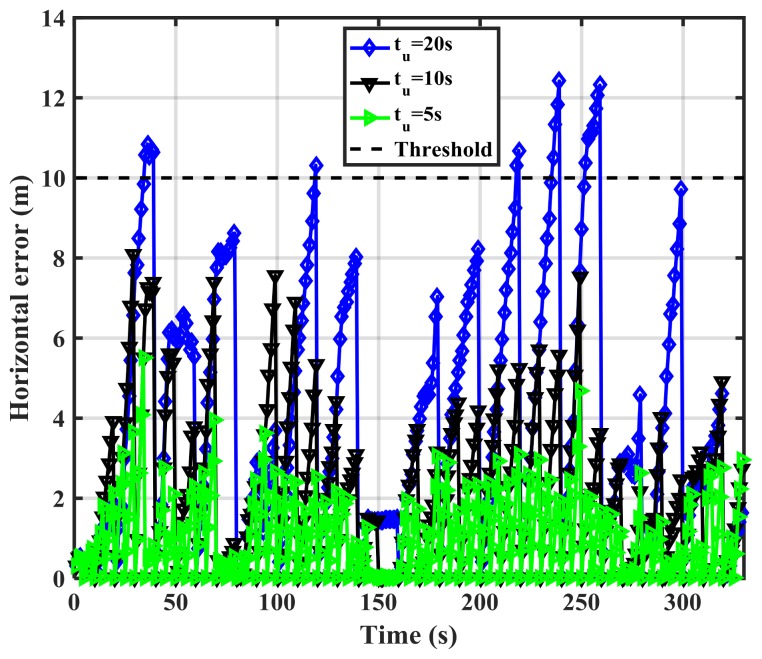
RIO (Cell phone IMU and vehicle odometer) horizontal errors for various update intervals (*t_u_*).

The update interval should be tuned to avoid false spoofing detection caused by the IMU sensor errors observed under the nominal operation conditions shown in Figure 5. The error pattern is a function of the IMU drift rate. Assuming correct initial RIO heading and position values, Figure 5 shows that both solutions meet the stated performance (horizontal errors below 10 m) during the first 30 s. Hence, one may compare the two solutions and correct the RIO errors every 30 s during the detection process. Figure 7 shows the MEMS IMU-based RIO errors for different update intervals (*t_u_*). The errors are the differences between authentic GPS and RIO solutions.

**Figure 8:**
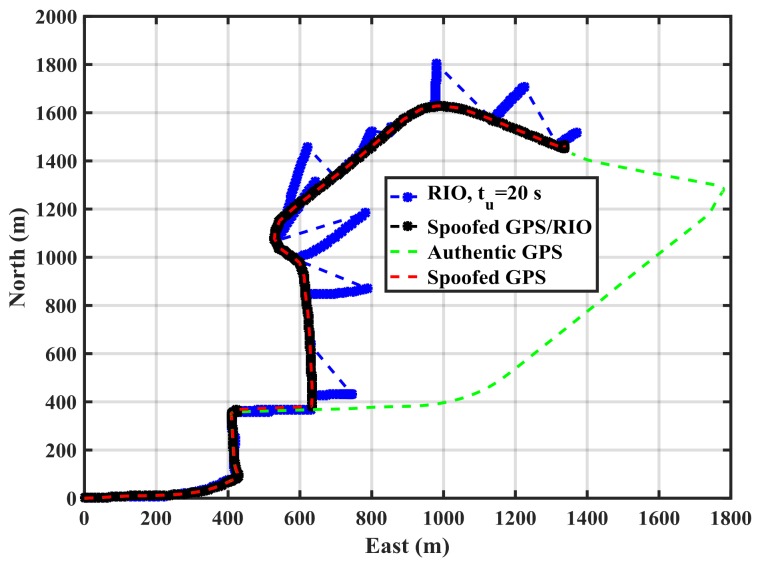
Horizontal trajectories for spoofed GPS, GPS/RIO, RIO with 20 s correction updates for MEMS (cell phone) IMU.

As expected, increasing the update interval increases the errors due to IMU drift. For *t_u_* = 20 s, the error values exceed 10 m at a few epochs. However, a longer observation period results in better detection performance as will be shown later. The above process is now implemented using the spoofed GPS red trajectory of Figure 4 by driving the green trajectory with the RIOs to assess spoofing detection effectiveness. Figure 8 shows the spoofed GPS trajectory, the spoofed GPS/RIO conventional integration trajectory in which case the RIO solutions are continuously corrected, and RIO solutions with correction intervals of *t_u_* = 20 s using the cellphone MEMS IMU and vehicle odometer. The actual authentic trajectory is also shown. The GPS/RIO trajectory remains spoofed and its trajectory matches the spoofed GPS one. This is because the Kalman filter puts more weights on normally high-quality GPS measurements than on the high drift MEMS IMU measurements. Nevertheless, the RIO solutions significantly deviate from the spoofed GPS and GPS/RIO trajectories, confirming spoofing detection, an important warning to the user.

**Figure 9:**
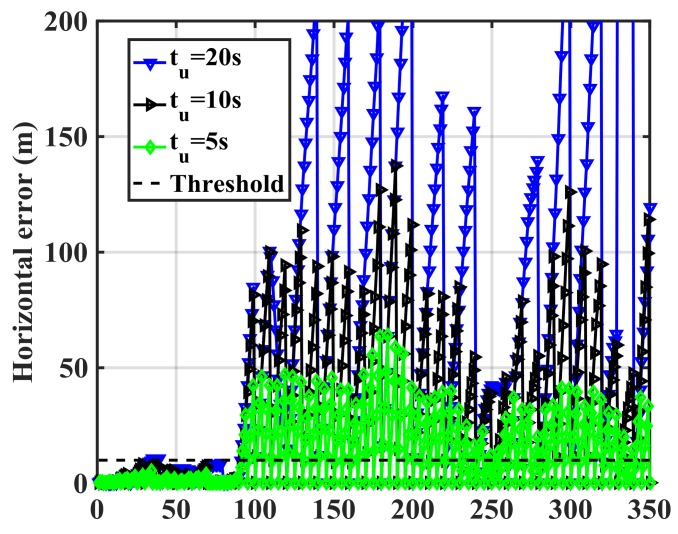
RIO horizontal errors for various update intervals (*t_u_*) in the spoofing case using the cellphone MEMS IMU.

Figure 9 shows the RIO errors for different update intervals (*t_u_*) in the spoofing case. The errors are the differences between GPS and RIO solutions. During the first 80 s, the receiver was not spoofed, hence spoofing detection metric outputs (error values) are below the threshold. However, once the spoofing attack begins, the errors significantly rise. As expected the longer the update interval, the larger the errors, hence better correct spoofing detection. Detection performance is a function of the relative authentic and spoofed trajectories and consequently the horizontal error values shown in the figure vary as a function of time. For instance, during the time interval 240–260 s, the horizontal error for *t_u_* = 5 s is below the detection threshold and the attack during this period cannot be detected. This is because the spoofing trajectory matches the authentic one during this period. In general, a spoofing attack using this approach cannot be detected during periods when GPS and RIO trajectories match.

### 5.2. Results of Data Set 2 (Dense Urban Environment)

**Figure 10:**
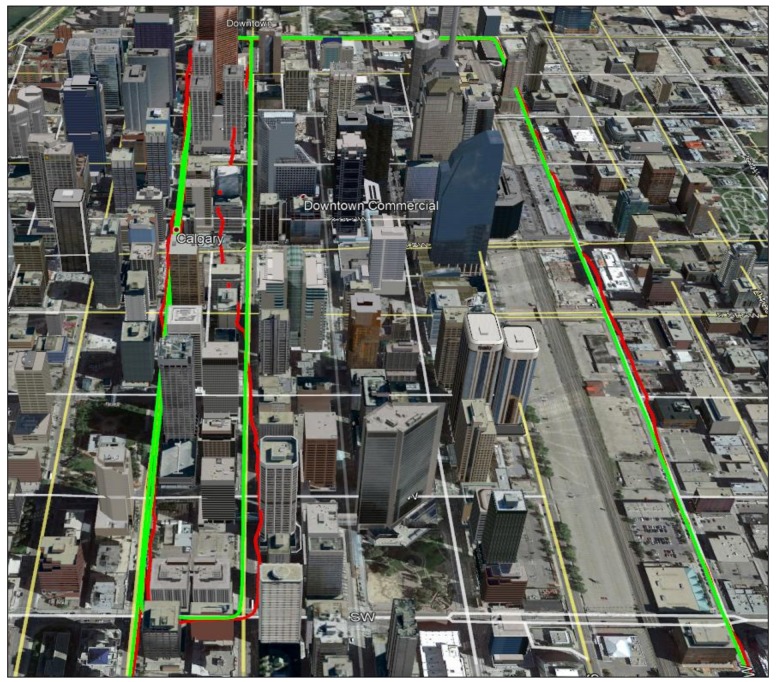
Test trajectory in dense urban environment with the correct reference trajectory (1–2 m accuracy) in green and the GPS trajectory in red. The latter is degraded due to multipath, high measurement noise and poor geometry.

Figure 10 shows the trajectory analysed in downtown Calgary, which is surrounded by buildings of up to 80 stories in height. The green and red plots show the correct reference and GPS-based trajectories. The reference trajectory was provided by the NovAtel SPAN system described earlier with accuracy of 1 to 2 m. The GPS trajectory was obtained with a u-blox receiver. In this dense area, the GPS trajectory deviates from the reference one by up to 50 m horizontally due to GPS multipath, high measurement noise and poor geometry. The data set includes repeated runs of the above trajectory. The authentic scenario refers to the case when the GPS and RIO trajectories are synchronized both in time and location. The spoofing case refers to the scenario where the GPS and RIO trajectories do not match either in time and/or in location; this is done by deliberately introducing a time lag between the GPS and RIO trajectory data. Different time lags (50 s to 200 s in 10 s intervals) were utilized to generate detection statistics.

**Figure 11:**
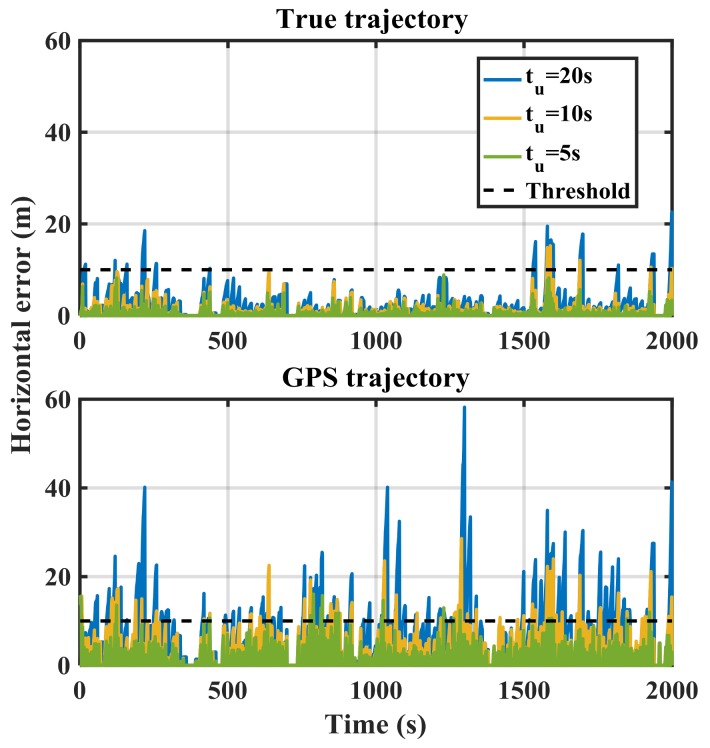
RIO (MEMS IMU) horizontal errors for various update intervals (*t_u_*) in the authentic case for true and GPS-based trajectories.

Figure 11 shows the RIO errors for MEMS IMU and different update intervals (*t_u_*) for the correct reference (green) and GPS-based (red) trajectories. The errors significantly increase in the GPS-based case due to multipath, measurement noise and geometry, which will increase spoofing detection false alarm probability. The error values of the true-reference trajectory shown in Figure 11 are similar to those of the GPS-based trajectory in open sky condition shown in Figure 7. To reduce a false alarm probability of the spoofing detection metric, one can increase the threshold but at the cost of reducing detection probability.

As expected, increasing the update interval increases the error values. For *t_u_* = 20 s, the errors exceed 10 m after a few epochs in the case of the true trajectory. However, threshold crossing happens in all *t_u_* cases in the GPS-based trajectory case. Poor GPS performance in dense urban environments affects the performance of the proposed spoofing detection metric in the null-hypothesis.

**Figure 12:**
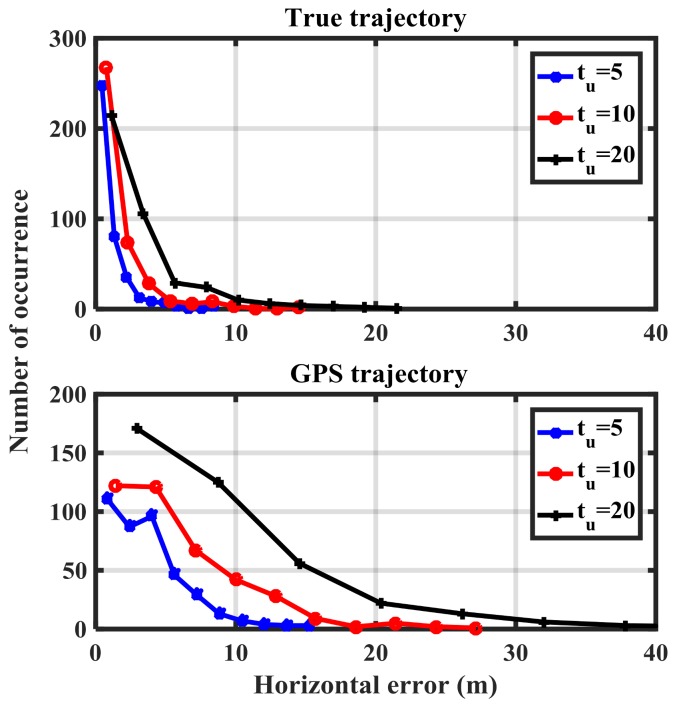
Histogram of the detection metric values for reference and GPS trajectories for different update intervals.

Figure 12 shows the histogram of the detection metric using true and GPS-based trajectories. This information will be used to determine the performance of the proposed method in terms of the receiver operation characteristics (ROC) plots.

**Figure 13:**
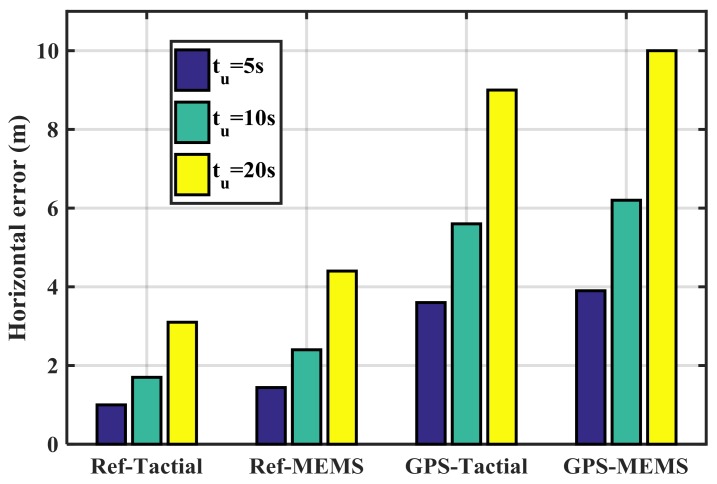
Mean trajectory errors for various combinations of GPS and IMU sensors for different update intervals.

Figure 13 shows the mean RIO errors for various combinations of GPS and IMU sensors in the authentic case. The Ref-Tactical case shown in Figure 13 refers to the true-reference trajectory and tactical IMU. This case has the lowest errors compared to other cases and causes the lowest probability of false detection. The worst performing scenario belongs to the GPS-MEMS case. In all the cases increasing the update interval (*t_u_*) increases the error values.

**Figure 14:**
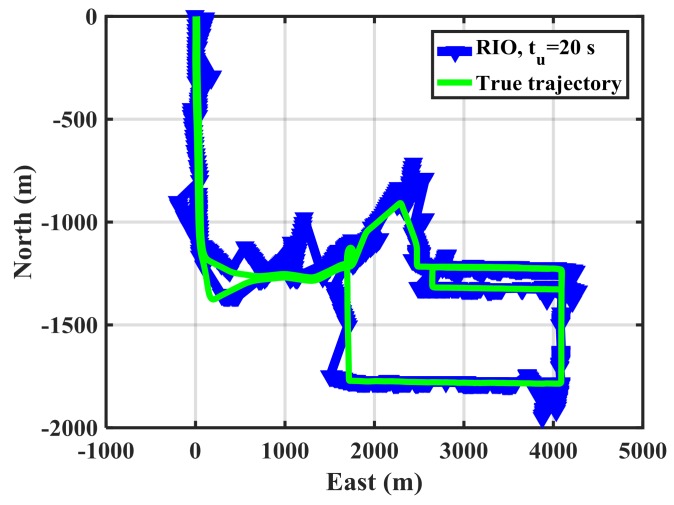
Horizontal trajectory for spoofed GPS, GPS/RIO, RIO with 20 s correction updates for MEMS IMU.

Figure 14 shows the horizontal trajectories for GPS and RIO solutions with *t_u_* = 20 s in the spoofing case. The RIO solutions significantly deviate from the GPS ones.

**Figure 15:**
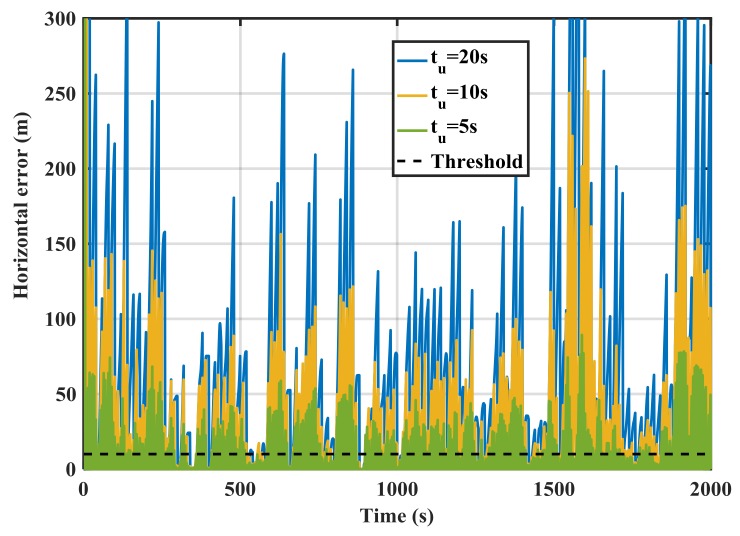
RIO horizontal error for various update intervals (*t_u_*) in the spoofing case for MEMS IMU.

Figure 15 shows the RIO mechanization errors for different update intervals (*t_u_*) in the spoofing case. The errors are the differences between GPS and RIO trajectories. The error values significantly rise compared to the results of Figure 11. As expected, increasing the update interval rises the error values and results in better detection performance.

**Figure 16:**
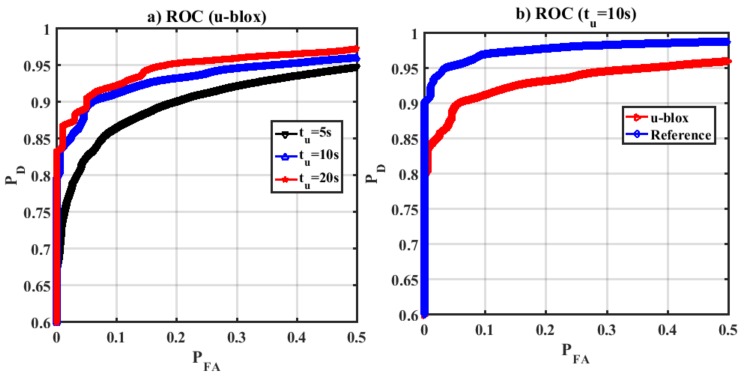
ROC curves (**a**) GPS-based trajectory (u-blox receiver) and different update intervals. Increasing *t_u_* improves the detection performance. (**b**) ROC curves for true-reference and GPS trajectories for *t_u_* = 10 s. The detection performance is degraded due to GPS measurement errors.

Receiver operation characteristics (ROC) which shows the performance of a detector was evaluated under different scenarios. Figure 16a shows ROC curves for the proposed detection metric for various update intervals using the u-blox GPS trajectory. Increasing *t_u_* enhances both probability of false alarm and detection. However, as shown, for a given false alarm probability, increasing *t_u_* enhances the detection performance. Figure 16b shows ROC plots for *t_u_* = 10 s for true-reference and GPS trajectories, demonstrating that spoofing detection performance is degraded in dense urban environments due to satellite geometry, high noise and multipath as mentioned earlier.

A missed detection probability is calculated if in each observation interval (*t_u_*), in the spoofing case, the detection metric is below the detection threshold. The measured missed detection rate for GPS-based trajectory for *t_u_* = 5 s, *t_u_* = 10 s and *t_u_* = 20 s is 21%, 7% and 2% respectively. As demonstrated increasing the observation period reduces the miss detection performance. The mean spoofing detection time for the given data set is 3.2 s, 3.4 s and 3.5 s for *t_u_* = 5 s, *t_u_* = 10 s and *t_u_* = 20 s cases respectively.

## 6. Conclusions

A GNSS authenticity verification approach based on integration of an IMU and a vehicle odometer outputs was proposed and tested. Contrary to conventional GNSS/INS coupling where the INS corrections are updated at each mechanization interval (mechanization and error correction rates are the same), the authenticity verification loop error correction rate in the present case is much lower than that of the mechanization process. The IMU/odometer navigation solutions can then be used to detect spoofing attacks at each correction interval. This approach is effective in detecting spoofing attacks, especially in covered spoofing scenarios when the authentic signals are blocked. For nominal operation scenarios and typical GPS and IMU sensors, a 20 s observation interval provided an acceptable detection performance in terms of probability of detection and mean time to detect the attack in the scenarios evaluated. Detection performance is a function of the relative authentic-spoofing trajectories; when the vehicle is static or travels in a straight direction with a constant speed, no feature can however be used to detect an attack. Some specific motion features such as continuous user velocity and heading changes (e.g., stops at traffic control lights and turns) provide additional features, resulting in better detection performance. Actual measurements in sub-urban and dense urban environments using different IMUs provided promising results to detect spoofing attacks in practical vehicular scenarios. GPS measurement quality affected by poor satellite geometry, signal attenuation and multipath occurring in urban canyons, degrades spoofing detection performance. In an actual implementation, these effects might be first detected using other metrics (e.g., C/N_0_) and then can be used to adjust the detection threshold. For the given data and spoofing scenario described in the papers, a mean spoofing detection time of 4 s was achieved.

## Figures and Tables

**Table 1:** IMU characteristics.

IMU	Parameter	Value
Tactical NovAtel SPAN IMU-LCI	Accelerometer bias	0.5 mg
	Accelerometer white noise	40 µg/√Hz
	Gyro drift	0.3°/h
	Gyro white noise	0.001°/s/√Hz
Cell-phone grade MEMS MPU 6500 (LG G3)	Accelerometer bias	60 mg max
	Accelerometer white noise	300 µg/√Hz
	Gyro drift	0.24°/s
	Gyro white noise	0.01°/s/√Hz

## Abbreviations

*DS*	Detection Statistics
$v_{i}^{o}$	speed from the odometer
$a_{i}^{o}$	acceleration from the vehicle odometer
$f_{i}^{x}$	x accelerometer outputs
$f_{i}^{y}$	y accelerometer outputs
$\omega_{i}^{z}$	vertical gyroscope output
$x_{k}$	Mechanization vector
$\left\{\varphi_{k},\lambda_{k},h\right\}$	position vector in the geodetic coordinate (latitude, longitude and height)
$\left\{v_{k}^{E},v_{k}^{N},v_{k}^{U}\right\}$	velocity vector in the East-North-Up (ENU) coordinate frame
$\left\{p_{k},r_{k},A_{k}\right\}$	pitch, roll and azimuth angles
g	gravity
$\delta x_{k}$	error state vector
$\delta S_{k}^{od}$	scale factor of odometer
$\delta \omega_{k}^{z}$	vertical gyroscope drift
$\Phi_{k-1}$	state transition matrix
$G_{k-1}$	shaping matrix or noise coupling matrix
$W_{k-1}$	zero mean unity variance white noise
$\gamma_{od}$	inverse of autocorrelation time for odometer stochastic errors
$\gamma_{\omega z}$	inverse of autocorrelation time for gyroscope stochastic errors
$\sigma_{od}^{2}$	variance of odometer noise
$\sigma_{\omega z}^{2}$	variance of gyroscope noise
$\delta z_{k}$	measurement vector
H	design matrix
$\varepsilon_{k}$	measurement noise
